# Hemodynamic Instability after Low-Energy Thigh Contusion Caused by Injury to the Femoral Artery: A Case Report and Literature Review

**DOI:** 10.1155/2016/9314297

**Published:** 2016-05-18

**Authors:** Juan Miguel Rodríguez-Roiz, José Ballesteros-Betancourt, Raquel García-Tarriño, Victor Antonio Rodríguez-Roiz, Manuel Llusa

**Affiliations:** ^1^Department of Orthopaedic Surgery and Traumatology, Hospital Clínic, C/Villarroel 170, 08036 Barcelona, Spain; ^2^Department of Orthopaedic Surgery and Traumatology, Hospital de l'Esperit Sant, C/Avinguda Mossen Pons i Rabadà s/n, Santa Coloma de Gramenet, 08923 Barcelona, Spain

## Abstract

Acute vascular injuries have been described in relation to high-energy trauma accidents or in patients undergoing surgery in the femoral area. We describe a healthy patient who sustained a direct, low-energy contusion in the thigh and presented haemodynamic instability. Arteriography was used to locate the point of bleeding, and embolisation and vessel occlusion were carried out to stop the haemorrhage. The genetic study identified the COL3A1 gene mutation; accordingly, the patient was diagnosed with the Ehlers-Danlos syndrome type IV (vascular type).

## 1. Introduction

Acute vascular injuries have been described in relation to high-energy accidents in patients who are polytraumatised or undergoing surgery in the femoral area [1–4], but this diagnosis is very rare in young patients with no relevant medical history. In such cases, clinicians should be alert to the possibility of structural alterations in the vessels that predispose them to injury or impaired blood clotting [5–9]. We present the case of a patient who sustained a direct, low-energy contusion in the thigh and presented a femoral injury. We also describe the treatment applied and the studies performed and review the relevant literature.

## 2. Case Presentation

**Figure 1:**
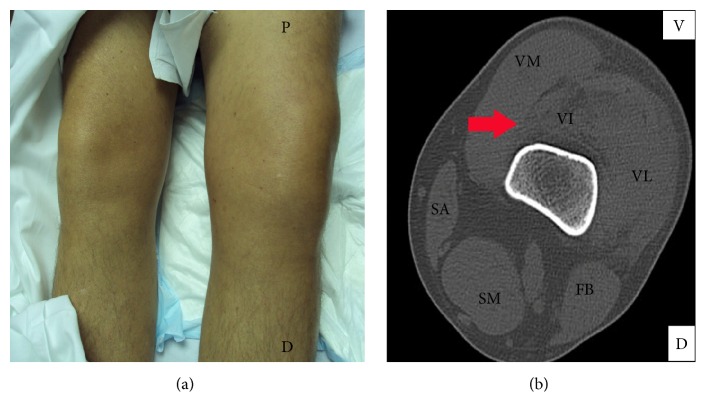
(a) Asymmetry can be observed between the two limbs due to swelling in the left thigh. P: proximal. D: distal. (b) The CT scan shows the presence of a perifemoral haematoma (indicated by the red arrow). The haematoma affects the vastus intermedius (VI) and a small image of active bleeding is visible in its interior. V: ventral. D: dorsal. VM: vastus medialis. VL: vastus lateralis. VI: vastus intermedius. SA: sartorius. FB: femoral biceps. SM: semimembranosus muscle.

**Figure 2:**
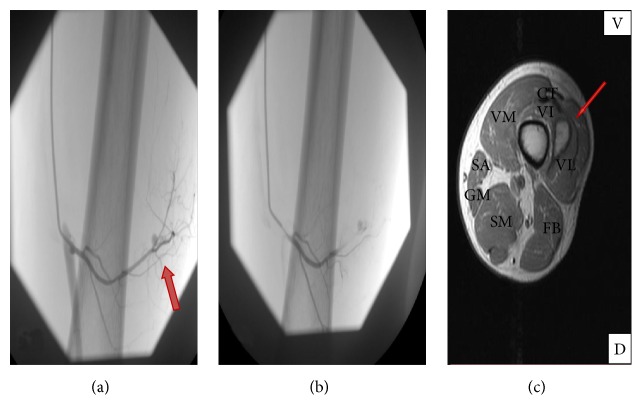
(a) A selective arteriography shows a point of active bleeding in a branch of the femoral artery at the level of the femoropopliteal junction (red arrow). (b) The image shows selective catheterisation of the bleeding branch (using Spongostan® particles) to carry out the correct occlusion of the branch. (c) Nuclear magnetic resonance (axial thigh T1) is performed after a month to monitor the patient's evolution. Subacute haematoma is observed in the distal third of the left thigh in the anterolateral compartment within the vastus intermedius muscle (VI). The haematoma did not present signs of spreading to other compartments or of affecting the adjacent femur. V: ventral. D: dorsal. VM: vastus medialis. VL: vastus lateralis. CT: quadriceps tendon. SA: sartorius. GM: gracilis muscle. FB: femoral biceps. SM: semimembranosus and semitendinosus muscle. Red arrow: subacute haematoma in resolution.

Forty-one-year-old male patient, with no known medical allergies or preexisting pathologies of interest except for a susceptibility to developing ecchymoses, presented at our emergency room. He complained of pain in the left thigh after fortuitously hitting the edge of a table the same morning while walking quickly. Physical examination revealed significant swelling in the anteromedial area of the right thigh at the level of the distal third (Figure 1(a)). While awaiting X-ray, the patient began to show additional symptoms including sweating, nausea, and increased pain. Given the presence of haemodynamic instability and the increase in the diameter of the thigh, an urgent CT scan was performed, which showed a haematoma affecting the vastus intermedius and gluteus muscles of the patient's left leg and a small image of active bleeding in the interior (Figure 1(b)). In view of the active bleeding in the thigh, it was decided to perform a selective arteriography, which identified the source of bleeding in a branch of the superficial femoral artery near the femoropopliteal junction. After selective catheterisation of the bleeding branch of the artery, embolisation was performed with particles of Spongostan (*Johnson & Johnson, Ferrosan Medical Services*,* Denmark*) until occlusion was achieved with arteriographic monitoring (Figures 2(a) and 2(b)).

During hospitalisation, the patient was kept haemodynamically stable and presented good evolution with gradual reduction of the haematoma as a result of rest, cryotherapy, and the administration of nonsteroidal anti-inflammatory drugs, achieving gradual functional recovery in the limb. The patient attended the outpatient service of the Orthopaedic Surgery and Traumatology unit, recovered total function of the limb, and resumed normal activities within a month of the contusion (Figure 2(c)). Evaluation requested from the Haemostasis Service in the hospital ruled out any kind of coagulopathy. However, the examination identified the COL3A1 gene mutation, and so the Ehlers-Danlos syndrome type IV (vascular type) was diagnosed. The patient was sent to the unit responsible for monitoring and controlling this disorder.

## 3. Discussion

Contusions of the musculoskeletal system are among the commonest causes of emergency-room consultation. Vascular injury should be suspected in all patients with a history of trauma, including non-high-energy trauma, when haemodynamic instability appears [1–4]. At this point, additional studies need to focus on locating the affected area, and the patient should be admitted to intensive care to ensure that there is no deterioration of vital signs. In some cases, a haematological study is recommended in addition to the genetic study to rule out disorders that may predispose the patient to similar clinical events [5–10]. In daily practice, however, the diagnosis of vascular injury of this type is extremely rare in young patients with no relevant medical history. In such cases, clinicians should look out for structural alterations in the vessels that predispose them to injury or impaired blood clotting. In patients with a history of collagen alterations (e.g., Ehlers-Danlos syndrome), weakness in the tissue of the large arteries, intestine, and uterus leads to a high predisposition to arterial rupture in any location either spontaneously or after mild trauma [5–9]. Ehlers-Danlos syndrome is a rare heterogeneous group of connective tissue disorders caused by a defect in the synthesis of collagen, with estimated population prevalence between 1/5000 and 1/25000 [11]. The vascular type, formerly known as Ehlers-Danlos-Syndrome type IV, affects 5–10% of all cases of this syndrome.

Life expectancy may be shortened by vascular Ehlers-Danlos syndrome. The median survival described in the literature varies between 48 and 50 years, due to the possibility of organ and vessel rupture. Approximately 25% of patients experience the first complication by the age of 20, while 80% have at least one complication by the age of 40 [12]. Although there is no way of preventing serious haemorrhagic accidents in these patients, identification of the condition is useful for indicating the best treatment. As far as possible, invasive treatment procedures should be avoided, as surgery can lead to vascular catastrophes and even death [11].
